# Concentration of Lead, Mercury, Cadmium, Aluminum, Arsenic and Manganese in Umbilical Cord Blood of Jamaican Newborns

**DOI:** 10.3390/ijerph120504481

**Published:** 2015-04-23

**Authors:** Mohammad H. Rahbar, Maureen Samms-Vaughan, Aisha S. Dickerson, Manouchehr Hessabi, Jan Bressler, Charlene Coore Desai, Sydonnie Shakespeare-Pellington, Jody-Ann Reece, Renee Morgan, Katherine A. Loveland, Megan L. Grove, Eric Boerwinkle

**Affiliations:** 1 Division of Epidemiology, Human Genetics, and Environmental Sciences (EHGES), University of Texas School of Public Health at Houston, Houston, TX 77030, USA; E-Mail: eric.boerwinkle@uth.tmc.edu; 2 Division of Clinical and Translational Sciences, Department of Internal Medicine, University of Texas Medical School at Houston, Houston, TX 77030, USA; 3 Biostatistics/Epidemiology/Research Design (BERD) Component, Center for Clinical and Translational Sciences (CCTS), University of Texas Health Science Center at Houston, Houston, TX 77030, USA; E-Mails: aisha.s.dickerson@uth.tmc.edu (A.S.D.); manouchehr.hessabi@uth.tmc.edu (M.H.); 4 Department of Child & Adolescent Health, The University of the West Indies (UWI), Mona Campus, Kingston 7, Jamaica; E-Mails: msammsvaughan@gmail.com (M.S.-V.); cooredesai@googlemail.com (C.C.D.); sydonniesp@gmail.com (S.S.-P.); jodyreece@yahoo.com (J.-A.R.); renee.n.morgan@gmail.com (R.M.); 5 Human Genetics Center, University of Texas School of Public Health at Houston, Houston, TX 77030, USA; E-Mails: jan.bressler@uth.tmc.edu (J.B.); megan.l.grove@uth.tmc.edu (M.L.G.); 6 Department of Psychiatry and Behavioral Sciences, University of Texas Medical School at Houston, Houston, TX 77054, USA; E-Mail: katherine.a.loveland@uth.tmc.edu

**Keywords:** lead, mercury, aluminum, arsenic, cadmium, manganese, cord blood, newborns, Jamaica

## Abstract

The objective of this study was to characterize the concentrations of lead, mercury, cadmium, aluminum, and manganese in umbilical cord blood of Jamaican newborns and to explore the possible association between concentrations of these elements and certain birth outcomes. Based on data from 100 pregnant mothers and their 100 newborns who were enrolled from Jamaica in 2011, the arithmetic mean (standard deviation) concentrations of cord blood lead, mercury, aluminum, and manganese were 0.8 (1.3 μg/dL), 4.4 (2.4 μg/L), 10.9 (9.2 μg/L), and 43.7 (17.7 μg/L), respectively. In univariable General Linear Models, the geometric mean cord blood aluminum concentration was higher for children whose mothers had completed their education up to high school compared to those whose mothers had any education beyond high school (12.2 μg/L *vs.* 6.4 μg/L; *p* < 0.01). After controlling for maternal education level and socio-economic status (through ownership of a family car), the cord blood lead concentration was significantly associated with head circumference (adjusted *p* < 0.01). Our results not only provide levels of arsenic and the aforementioned metals in cord blood that could serve as a reference for the Jamaican population, but also replicate previously reported significant associations between cord blood lead concentrations and head circumference at birth in other populations.

## 1. Introduction

Exposure to several trace elements/heavy metals, including lead, mercury, cadmium, arsenic, and aluminum during pregnancy has been shown to be harmful to the developing fetus [1,2,3,4,5,6,7,8,9] and can be harmful to the human nervous system, even at low levels of exposure [10,11,12,13]. The adverse effects of lead exposure during pregnancy on birth outcomes have been well documented, including lower birth weight [14], lower birth crown-heel length and head circumference [15,16,17], and preterm birth [18,19]. Also, several studies have reported that methyl mercury could easily cross the placenta and affect cognitive development [3,4,20,21,22]. Although the placenta acts as a barrier, protecting the fetus from cadmium exposure by increasing metallothionein expression [23], the presence of cadmium in cord blood has been associated with decreased birth weight [24] and increased incidence of preterm delivery [25].

Sources of exposure to lead include leaded gasoline, leaded paint, dust and soil contaminated with lead, water carried in lead pipes, industrial emissions or occupational exposures [26]. Exposure to mercury mainly occurs through ingestion of contaminated fish (methyl mercury) [27] or dental amalgams (inorganic mercury) [28]. Environmental cadmium pollution is ubiquitous owing to industrial activities, use of phosphate fertilizers, combustion of motor fuels in vehicles and particles released by tire wear, all of which result in emissions to air, soil and water [29,30,31]. Although smoking tobacco is the most important source of exposure to cadmium, in nonsmokers diet is considered as the most important source of cadmium exposure [32]. A study that investigated the role of air pollutants in birth outcomes has reported statistically significant associations between low birth weight and levels of aluminum (in all trimesters), calcium, nickel, silicon, and zinc (in the third trimester), as well as elemental carbon and titanium (in the first trimester) [33,34]. Shi *et al.* reported preterm birth has a stronger spatial association with groundwater arsenic than term low birth weight in New Hampshire, USA [35]. Manganese is an essential mineral nutrient that plays an important role in fetal development and in other important aspects of metabolism. However, elevated manganese can have potential neurotoxic effects, particularly in infants. Limited information is available regarding the effects of elevated manganese in the development of the human fetus. The findings from a relatively recent study indicate that lower maternal blood manganese is associated with fetal intrauterine growth retardation and lower birth weight [36]. Although, a study based on a cohort of 470 mother-infant pairs from Oklahoma did not find a significant linear association between umbilical cord blood manganese and infant birth weight, they did find a non-linear relationship between maternal blood manganese and infant birth weight [37].

Previous studies have reported a high level of several trace elements and heavy metals in the soil of Jamaica [38] including lead [38,39], aluminum [38,40], arsenic [38], and cadmium [41,42]. Wright *et al.* investigated concentrations of nine residual metals (chromium, manganese, nickel, copper, zinc, arsenic, cadmium, lead, and mercury) in some Jamaican foods and reported a significant correlation (*r* ≥ 0.7) between levels of lead in soil and agricultural produce [43]. However, Howe *et al.* conducted a similar study in Jamaica and reported that lead absorbed by vegetables did not pose a significant risk of elevated blood lead concentrations in the Jamaican population [44]. Recent reports indicate that the arithmetic mean blood arsenic concentration of Jamaican children is 4.55 μg/L [45] and is about 4.5 times the blood arsenic concentration for unexposed individuals in the US [46]. Similar reports from our research in Jamaica also indicate that the arithmetic mean blood cadmium concentration is relatively low, 0.16 μg/L [47], and that the arithmetic mean blood manganese concentration (10.9 μg/L) [48] and the arithmetic mean blood mercury concentration (1.16 μg/L), are the same as or more than three times higher than that of children from the US, respectively [49].

In this study, concentrations of the aforementioned trace elements/heavy metals in umbilical cord blood of Jamaican newborns were determined and factors associated with levels of these toxins were identified. In addition, possible relationships between cord blood metals and certain birth outcomes including birth weight, crown-heel length, and head circumference were explored.

## 2. Materials and Methods

### 2.1. General Description

Data for this study were generated in collaboration with faculty at the University of the West Indies (UWI), Mona Campus, Kingston, Jamaica. The JA Kids study, a birth cohort study in Jamaica, enrolled mothers between the third trimester and delivery in 2011 [50,51,52,53]. The participants in the study reported here comprise a subsample of 100 mothers with 100 newborns from whom cord blood was isolated. To the extent possible, data from two questionnaires administered to mothers in their third trimester and at the time of delivery by the JA Kids study were linked with concentrations of the aforementioned trace elements/metals. Through these two questionnaires, demographic and socioeconomic information, including level of maternal education, and certain assets owned by the family were collected. The mean age of mothers at delivery was 29.6 years. Fifty-five percent of the newborns were female. Sixty percent of mothers reported previous pregnancies. Information regarding other socio-demographic and socio-economic characteristics of the study population is provided in Table 1.

Study data were collected and managed using REDCap electronic data capture tools hosted at the University of Texas Health Science Center at Houston [54]. Also, 2 mL of cord blood were collected into plastic tubes containing EDTA which were prescreened for several trace elements or metals, including lead, mercury, arsenic, cadmium, manganese, and aluminum [55,56,57]. The blood samples were frozen and stored at −20 °C until they were transported to the Michigan Department of Community Health (MDCH) Trace Metals Lab at ambient temperature on ice packs for trace metal analyses. Institutional Review Boards (IRBs) of the University of Texas Health Science Center at Houston and UWI approved this study. All participating mothers provided written informed consent, in compliance with the IRBs. The data presented herein represent analysis of 100 singleton newborns for whom we had complete data.

**Table 1:** Individual and household characteristics of the study sample.

Variables	Categories	N (%)
Sex of newborn	Male	45 (45%)
	Female	55 (55%)
Maternal age (years) (at newborn’s birth)	Age < 25	21 (21%)
	25 ≤ age < 30	34 (34%)
	Age ≥ 30	45 (45%)
Maternal education (at newborn’s birth)	Up to high school	30 (30%)
	Beyond high school	70 (70%)
Previous pregnancies ^a^	Yes	57 (60%)
	No	38 (40%)
Assets owned by the Family	TV ^b^	99 (100%)
	Refrigerator ^c^	98 (99%)
	Freezer ^d^	30 (37%)
	Living room set ^e^	80 (86%)
	Washing machine ^f^	75 (81%)
	Cars or other vehicle	51 (51%)
	Cable/Satellite connection ^g^	72 (74%)

^a^ Previous pregnancies is missing for 5 mothers; ^b^ TV ownership is missing for 1 family; ^c^ Refrigerator ownership is missing for 1 family; ^d^ Freezer ownership is missing for 18 families; ^e^ Living room set ownership is missing for 7 families; ^f^ Washing machine ownership is missing for 7 families; ^g^ Cable/Satellite connection is missing for 3 families .

### 2.2. Assessment of Lead, Mercury, Arsenic, Cadmium, Manganese, and Aluminum in Cord Blood

In this study, whole cord blood samples were assayed for lead, mercury, arsenic, cadmium, manganese, and aluminum by the Trace Metals Lab at MDCH, a CDC certified lab for analysis of trace metals. MDCH followed a fully validated protocol for analyzing lead, mercury, arsenic, cadmium, manganese, and aluminum in cord blood samples with limits of detection (LoD) of 0.25 μg/dL, 0.25 μg/L, 0.13 μg/L, 0.13 μg/L, 2.50 μg/L, and 5.0 μg/L, respectively. Ninety-nine percent of blood cadmium concentrations and 78% of blood arsenic concentrations were below the LoD. All samples were diluted and analyzed on a PerkinElmer Elan DRC II inductively-coupled plasma mass spectrometer (PerkinElmer, Waltham, MA, USA).

### 2.3. Statistical Analysis

The distributions of various characteristics of the study sample including demographic and socioeconomic status (SES) were examined. The distributions of various trace elements/metals in cord blood were also examined. If the distribution of any of the six trace elements/metals was skewed, the data were transformed using the natural logarithm (ln) in order to produce a distribution that better approximated a normal distribution. Descriptive statistics were used to characterize the distribution of the six trace elements/metals, including geometric means and standard deviations [58,59] for the ln-transformed variables (*i.e.*, lead, mercury, and aluminum) and arithmetic means and standard deviations for all six of the trace elements/metals. Other descriptive measures are also provided, including median, interquartile range, and 25th, 75th, 90th, and 95th percentiles. For concentrations below the LoD, measurements were imputed by the midpoint between zero and the LoD for each trace element/metal.

General Linear Models (GLMs) were used to evaluate significant associations between blood trace element/metal concentrations and various socio-demographic characteristics of mothers and their newborns, including maternal age at the time of delivery, maternal education level, and SES as assessed by car ownership. In addition, possible associations of cord blood concentrations of lead, mercury, aluminum, and manganese with birth outcomes, including birth weight, crown-heel length, and head-circumference were assessed. Multivariable linear regression models were also fit to control for potential confounding by maternal education level and SES while investigating the associations between cord blood concentrations of the aforementioned trace elements/metals and birth outcomes. Since a majority of cord blood metal concentrations for arsenic and cadmium were below the LoD, the latter analyses did not include these two metals. All descriptive and inferential statistical analyses were conducted using SAS 9.3 [60]. All statistical tests were conducted at 5% level of significance.

## 3. Results

The arithmetic mean (standard deviation (SD)) concentrations of cord blood lead, mercury, aluminum, and manganese in the sample of newborns in this study were 0.8 (1.3 μg/dL), 4.4 (2.4 μg/L), 10.9 (9.2 μg/L), and 43.7 (17.7 μg/L), respectively. Because lead, mercury, and aluminum concentrations were not normally distributed, geometric mean (SD) blood concentrations of 0.6 (1.8 μg/L), 3.9 (1.6 μg/L), and 7.7 (2.4 μg/L) were also calculated. Notably, the percentage of cord blood samples with metal levels below the LoD was 2% for lead, 30% for aluminum, 99% for cadmium, and 78% for arsenic, and 0% for manganese and mercury. Additional information on the distributions of the six trace elements/heavy metals is displayed in Table 2.

**Table 2:** Distribution of lead, mercury, arsenic, cadmium, manganese, and aluminum concentrations in umbilical cord blood in a sample of *n* = 100 newborns from Jamaica.

Variables	N	Mean (SD)		Median	Interquartile Range	Percentiles			
		Arithmetic	Geometric			25th	75th	90th	95th
Lead (µg/dL)	100	0.8 (1.3)	0.6 ^a^ (1.8) ^b^	0.6	0.3	0.4	0.8	1.2	1.7
Mercury (µg/L)	100	4.4 (2.4)	3.9 ^a^ (1.6) ^b^	4.0	2.4	2.8	5.2	7.0	7.6
Aluminum (µg/L)	100	10.9 (9.2)	7.7 ^a^ (2.4) ^b^	8.6	13.0	2.5	15.5	22.5	27.5
Manganese (µg/L)	100	43.7 (17.7)	NR ^e^	41.0	21.0	41.0	52.0	66.5	80.0
Arsenic (µg/L) ^c^	100	1.0 (0.9)	NR ^e^	0.6	0.0	0.7	0.7	2.1	2.8
Cadmium (µg/L) ^d^	100	0.07 (0.01)	NR ^e^	0.07	0.00	0.07	0.07	0.07	0.07

^a^ Mean lead, mercury, and aluminum indicates the geometric mean = Exp. [Mean (ln of metal concentration)]; ^b^ SD of lead, mercury, and aluminum indicates the geometric standard deviation = Exp. [standard deviation of (ln of metal concentration)]; ^c^ 78 (78%) samples were below the limit of detection for arsenic; ^d^ 99 (99%) samples were below the limit of detection for cadmium. Also, for ease of understanding, the descriptive statistics for this variable are provided with two decimal places; ^e^ NR = geometric mean and standard deviation were not reported because ln-transformation was not applied to these variables.

The mean (SD) birth weight for Jamaican newborns in this sample was 3.1 (0.6 kg). The mean crown-heel length for the total sample was 46.7 (5.0 cm). Additionally, the mean head circumference of newborns was 33.5 (2.9 cm). The mean gestational age was 39.3 (5.9) weeks. Details about birth outcomes, stratified by the sex of the newborns, are presented in Table 3.

**Table 3:** Descriptive analysis of continuous birth outcome variables stratified by sex of newborn.

Birth Outcome Variables	N			Mean (SD)			Median Total	Interquartile Range Total
	Male	Female	Total	Male	Female	Total		
Head circumference (cm)	25	30	55	34.0 (1.3)	33.1 (3.7)	33.5 (2.9)	34.0	2.0
Crown-heel length (cm)	22	26	48	46.6 (5.3)	46.8 (4.8)	46.7 (5.0)	48.0	3.5
Birth weight (Kg)	44	52	96	3.1 (0.6)	3.0 (0.5)	3.1 (0.6)	3.1	0.6
Apgar 1-minute scores	34	43	77	7.9 (1.7)	8.4 (1.0)	8.2 (1.4)	9.0	1.0
Gestational age (weeks)	35	38	73	38.0 (2.2)	40.5 (7.8)	39.3 (5.9)	39.0	2.0

In univariable GLMs, the geometric mean blood aluminum concentration was higher for newborns whose mother did not have education beyond high school compared to those whose mother had attained this level (12.2 *vs.* 6.4 μg/L; *p* < 0.01). In addition, newborns who belonged to a higher SES group (*i.e.*, family owned a car) had a lower geometric mean blood aluminum concentration than newborns in families who did not own a car (6.4 *vs.* 9.3 μg/L; *p* = 0.03). Other associations between exposure to trace elements/metals and mothers’ individual and household characteristics are presented in Table 4. Correlation analysis of cord blood metal concentrations with birth outcomes revealed that cord blood lead concentration was significantly correlated with birth head circumference (*p* < 0.01).

**Table 4:** Univariable association of cord blood metal concentrations with mother’s individual and household characteristics as potential confounding variables based on data from *n* = 100 newborns.

Variables	Category	N	Lead		Mercury		Aluminum		Manganese	
			Mean ^a^ (SD) ^b^	*p*	Mean ^a^ (SD) ^b^	*p*	Mean ^a^ (SD) ^b^	*p*	Mean (SD)	*p*
Maternal age (years) (at newborn’s birth)	<30	55	0.6 (2.6)	0.53	3.7 (1.9)	0.22	8.4 (3.3)	0.27	44.0 (24.3)	0.83
	≥30	45	0.7 (2.4)		4.2 (2.1)		7.0 (3.7)		43.2 (26.9)	
Maternal education (at newborn’s birth)	Up to high school	30	0.6 (3.2)	0.88	3.7 (2.4)	0.35	12.2 (4.5)	<0.01	42.8 (32.9)	0.74
	Beyond high school	70	0.6 (2.1)		4.1 (1.8)		6.4 (2.7)		44.1 (21.5)	
Previous pregnancies ^c^	Yes	57	0.6 (2.4)	0.51	3.9 (1.9)	0.55	8.0 (3.2)	0.81	43.0 (24.2)	0.56
	No	38	0.6 (2.8)		4.1 (2.4)		7.7 (4.1)		45.2 (29.7)	
Car ownership	No	49	0.6 (2.5)	0.73	3.8 (2.0)	0.30	9.3 (3.4)	0.03	46.0 (25.5)	0.21
	Yes	51	0.6 (2.4)		4.1 (2.0)		6.4 (3.3)		41.5 (25.0)	

^a^ Mean lead, mercury, and aluminum indicates the geometric mean = Exp. [Mean (ln of metal concentration)] ^b^ SD of lead, mercury, and aluminum indicates the geometric standard deviation = Exp. [standard deviation of (ln of metal concentration)] ^c^ Previous pregnancies is missing for 5 mothers.

**Table 5:** Correlation coefficients (r) between cord blood metal concentrations and birth outcome variables.

Birth Outcome Variables	N	Lead ^a^		Mercury ^a^		Aluminum ^a^		Manganese	
		r	*p*	r	*p*	r	*p*	r	*p*
Birth weight (kg)	96	<0.01	0.96	0.13	0.20	0.01	0.89	0.09	0.40
Crown-heel length (cm)	48	0.05	0.71	−0.07	0.64	0.04	0.79	−0.08	0.59
Head circumference (cm)	55	−0.74	<0.01	−0.13	0.35	−0.13	0.69	−0.18	0.20
Apgar 1-minute scores	77	−0.07	0.55	0.01	0.93	−0.10	0.40	0.01	0.93
Gestational age (weeks)	73	0.02	0.88	−0.03	0.78	−0.17	0.14	<0.01	0.96

^a^ For lead, mercury, and aluminum, ln-transformed cord blood metal concentrations were used.

No other metal concentrations were found to be significantly correlated with birth outcomes. Correlation coefficients for these analyses are reported in Table 5. In multivariable GLMs, after controlling for potential cofounding variables that included maternal education level and SES, no significant association of mercury, aluminum, and manganese concentrations in cord blood with any of the aforementioned birth outcomes was found. However, cord blood lead concentration was inversely associated with head circumference (adjusted *p* < 0.01). Moreover, though not statistically significant, the adjusted regression coefficients of head circumference with other cord blood metal concentrations were all negative. Adjusted slope coefficients and accompanying adjusted *p-*values for all multivariable models are shown in Table 6.

**Table 6:** Multivariable associations between cord blood metal concentrations and birth outcomes after controlling for SES and maternal education in the final multivariable linear regression models for birth outcomes.

Birth Outcome Variables	N	Lead ^a^		Mercury ^a^		Aluminum ^a^		Manganese ^b^	
		Adjusted Slope Coefficient	*p*	Adjusted Slope Coefficient	*p*	Adjusted Slope Coefficient	*p*	Adjusted Slope Coefficient	*p*
Birth weight (kg)	96	0.01	0.91	0.16	0.18	0.01	0.87	0.002	0.53
Crown-heel length (cm)	48	0.48	0.68	−1.31	0.47	−0.15	0.87	−0.013	0.79
Head circumference (cm)	55	−2.30	<0.01	−0.90	0.34	−0.28	0.59	−0.034	0.19
1-minute Apgar score	77	−0.15	0.56	−0.011 ^c^	0.98	−0.14	0.50	<0.001 ^d^	0.92
Gestational age (weeks)	73	0.30	0.77	−0.24	0.87	−1.46	0.10	−0.006	0.87

^a^ For lead, mercury, and aluminum ln-transformed cord blood metal concentrations were used; ^b^ For ease of understanding, the adjusted slope coefficients for manganese are provided with three decimal places; ^c^ For ease of understanding, the adjusted slope coefficient for 1-min Apgar score in relation to mercury is provided with three decimal places; ^d^ The actual value was 0.00097.

## 4. Discussion

### 4.1. Cord Blood Trace Element/Heavy Metal Concentrations in Jamaican Newborns

In this paper, information was provided on blood trace element/metal concentrations in a sample of 100 newborn cord blood samples from 100 pregnant women in Jamaica, which could serve as a reference for the Jamaican population. In the following, findings for each of the six trace elements/metals investigated in this study are discussed.

### 4.2. Lead Concentrations in Cord Blood

The cord blood arithmetic mean lead concentrations reported in this study is 0.8 (SD = 1.3 μg/dL). Although there are no previous reports regarding levels of cord blood lead concentrations in Jamaican newborns, studies from several other countries have reported such levels in different populations. For example, a study from Belgium reported cord blood arithmetic mean lead concentration of 1.5 μg/dL [61]. Two separate studies from France reported cord blood arithmetic mean lead concentrations of 1.5 μg/dL [62,63] and 2.3 μg/dL [62,63]. A study from Canada reported cord blood arithmetic mean lead concentration of 2.8 μg/dL [64], and another study from Saudi Arabia reported cord blood arithmetic mean lead concentration of 2.5 μg/dL [65]. These data suggest that cord blood lead concentrations in Jamaican newborns are lower than that of the aforementioned developing and developed countries.

### 4.3. Mercury Concentrations in Cord Blood

The geometric mean cord blood total mercury concentration in the sample of newborns in this study was 3.9 (SD = 1.6 μg/L). About 20% (21%) of cord blood mercury concentrations exceeded 5.8 μg/L, a level that has been shown to be associated with possible health effects in humans [66]. While these levels are higher than the geometric mean cord blood level reported in a community with high fish consumption in the Canadian Arctic (mean = 2.7 μg/L) [64] and a sample from Saudi Arabia (mean = 2.6 μg/L), these levels are lower than total mercury cord blood levels reported from Hawaii (mean = 5.2 μg/L) [67] and Spain (geometric mean total mercury = 8.2 μg/L) that are mainly attributed to maternal consumption of fish species high in mercury [68], while a study in an urban area in Spain only reported a geometric mean cord blood mercury concentration of 6.7 μg/L [69]. A previous report from our research in Jamaica also documented a significant association between seafood and fish consumption and mercury concentration in the blood of Jamaican children, aged 2–8 years [49]. Seafood, particularly due to the omega-3 polyunsaturated fatty acids in fish, is considered beneficial for human health [70]. While pregnant women are encouraged to eat fish during pregnancy because of its benefits, they are advised to limit its consumption due to contaminants such as methyl mercury in fish that may pose a hazard to the fetus [71]. Previous studies have reported conflicting findings regarding the association between total mercury concentrations in cord blood and birth weight. Some reported an inverse association between total mercury concentrations in cord blood and birth weight [72,73] while others reported no such association [74,75]. Considering that fish consumption is an important source of exposure to mercury, which could have both beneficial and potentially harmful effects, it would be difficult to provide evidence-based guidelines for seafood consumption for use by the public without conducting a risk-benefit analysis [76,77]. However, the State of Pennsylvania in the US provided an advisory for fish consumption in 2012 that guides the public regarding frequency of fish consumption [78]. Australia and New Zealand have jointly published guidelines for seafood consumption [79].

### 4.4. Cadmium Concentrations in Cord Blood

Ninety nine percent (99%) of cord blood samples had cadmium concentrations below the LoD of 0.13 μg/L. Nearly similar, a study in the Canadian Artic reported only 26% of cord blood samples obtained had detectable levels of cadmium [64]. This is not surprising because it has been documented that the placenta acts as a barrier against the passage of this metal [23]. In addition, our previous report from Jamaica documented that about 66% of our sample of typically developing children (aged 2–8 years) had blood cadmium concentrations that were below the LoD [47]. Considering that cigarette smoke is a well-known source of human exposure to cadmium [64,80,81], and it has been previously reported that 10% of mothers were smokers [82], the low observed cadmium blood concentrations are not surprising. However, notably, a study of healthy, non-smoking women reported that cord blood cadmium concentrations were associated with a decrease in birth weight [83].

### 4.5. Arsenic Concentrations in Cord Blood

In our sample, 78% of cord blood samples had arsenic concentrations below the LoD of 0.13 μg/L. However, a study that involved 460 newborns from Taiwan reported that no cord blood arsenic levels were below their LoD of 0.7 μg/L [84], while another study from China also indicated that none of the 2010 cord blood samples assessed were below their LoD of 0.41 μg/L [85]. Additionally, a study of 94 newborns from Nepal reported cord blood arsenic concentrations of at least 0.5 μg/L [86]. Our findings indicate that the cord blood arsenic concentrations in Jamaican newborns are much lower than those in other developing countries such as Taiwan and China.

### 4.6. Manganese Concentrations in Cord Blood

The cord blood manganese concentrations reported in this study (*i.e.*, arithmetic mean = 43.7 μg/L) are slightly higher than those observed in newborns from some parts of the developed world, including Paris, France (geometric mean = 38.5 μg/L) [87], other metropolitan cities in France (arithmetic mean = 31.2 μg/L) [62], and Canada (arithmetic mean = 34.4 μg/L) [88]. However, the cord blood manganese concentrations from our Jamaican sample are comparable to those of the US (arithmetic mean = 44.0 μg/L and 42.0 μg/L from two different studies) [37,89]. Additionally, a study from Tehran, Iran, reported cord blood concentrations of 44.7 μg/L and 38.2 μg/L in newborns with intrauterine growth restriction and appropriate growth while also demonstrating greater manganese concentrations for smaller newborns [90]. However, mean cord blood manganese concentrations of our Jamaican sample are lower than those of a sample of newborns from Taiwan who had a mean cord blood manganese concentration of 52.8 μg/L [84]. The mean blood manganese concentration of typically developing children (aged 2–8 years) in Jamaica has been reported in our previous results as lower than that of other developing countries, but comparable to that of children in more developed countries, including the US and Australia [48]. Similar to our results, a study from Shanghai, China, showed no significant relationship between cord blood serum manganese concentrations and gestational age or birth weight [91]. Another study from the US also did not find a statistically significant association between cord blood manganese concentrations and birth weight [37]. Although neurobehavioral development of infants was not assessed in our study, it is important to note that the study in China found that cord blood serum manganese concentrations were associated with poor neurodevelopment in neonates [91].

### 4.7. Aluminum Concentrations in Cord Blood

The arithmetic mean cord blood aluminum concentration in this study was 10.9 (SD = 9.2 μg/L) and is higher than that (arithmetic mean 8.5 μg/L = 0.04 μmol/L) reported in newborns from Rzeszów, Poland [92]. Previous studies have shown that there is no safe level for aluminum [93,94]. Furthermore, the soil of Jamaica contains several trace elements and heavy metals, including aluminum [38,44]. As an exporter of bauxite, Jamaica has important sources of exposure to aluminum [40,94,95], which supports our findings reported here.

### 4.8. Role of Cord Blood Metal Concentrations in Birth Outcomes

In this study, possible associations of umbilical cord blood concentrations of lead, mercury, manganese, and aluminum with several birth outcomes that included birth weight, crown-heel length, and head circumference were also investigated. Consistent with univariable analyses, after controlling for potential cofounding variables that included maternal education level and SES, in our multivariable analysis, cord blood lead concentration was significantly associated with head circumference (adjusted *p* < 0.01). However, a significant association of mercury, aluminum, and manganese concentrations in cord blood with any of the aforementioned birth outcomes was not found. Other studies, have reported associations of cord blood lead levels with birth outcomes such as birth weight [14,24,36,83,92,96], and head circumference [15,16,17]. However, investigations evaluating the possible effects of in utero exposure to mercury [97,98] and manganese [37,91] on fetal growth have reported conflicting findings. Specifically, some reported an inverse association between cord blood mercury and fetal growth [99,100] or Apgar scores [101]. Other studies including our findings in this report did not show any significant associations between cord blood mercury and the aforementioned birth outcomes [98,102]. Some reports have attributed this conflict to maternal fish consumption habits, as many species contain beneficial nutrients that can compensate for the toxic effects of mercury [99]. Since we did not have dietary information from mothers, potential confounding of the results observed in our study by type of fish consumed could not be evaluated. Overall, our findings do not support an association between total mercury in cord blood and birth outcomes. Although previous studies have reported that in utero exposure to cadmium may have detrimental effects on newborns’ health [24,25,103,104,105,106], in our study, such effects were not evaluated due to extremely low levels of exposure (*i.e.*, 99% were below the LoD).

### 4.9. Role of Maternal Education and SES in Cord Blood Metal Concentrations

In the process of identifying potential confounding variables, significant associations of maternal education level and socioeconomic status (measure by ownership of a car by the family) with cord blood aluminum concentrations were found. In the following, we discuss these auxiliary findings.

Our findings indicated higher geometric mean cord blood aluminum concentrations for newborns whose mothers did not have education beyond high school compared to those whose mothers had attained this level (12.2 *vs.* 6.4 μg/L; *p* < 0.01). Other studies have not reported significant associations between maternal education and cord blood heavy metal concentrations. For example, Amaya *et al.* (2013) investigated potentially associated maternal factors (such as maternal education) with prenatal exposure to arsenic, cadmium, mercury, manganese, and lead in a population-based mother–child cohort in Southern Spain and found gestational age and smoking during pregnancy were associated with placental cadmium concentrations, while no factors were significantly associated with mercury, manganese, or lead concentrations [107]. In contrast, a study from the UK reported that high education attainment was independently associated with higher blood lead levels in pregnant women [108]. In addition, our findings indicated that newborns who belonged to families who owned a car (*i.e.*, higher SES) had a lower geometric mean cord blood aluminum concentration than newborns in families who did not own a car (6.4 *vs.* 9.3 μg/L; *p* = 0.03). To our knowledge, no other studies have reported a significant association between socioeconomic status and cord blood aluminum concentrations, however, some studies reported that children with lower SES have higher exposures to many chemical contaminants [109]. For example, Ahamed *et al.* reported that children with the lowest SES status who lived in Lucknow, India had significantly higher blood lead concentrations compared with other SES levels (low = 12.0 μg/dL, middle = 8.4 μg/dL, and high = 5.8 μg/dL) [110]. In contrast, Rahbar *et al.* did not find a significant difference between the geometric mean blood lead concentration in Jamaican children from families who owned a car (*i.e.*, higher SES) and those who did not (*i.e.*, lower SES), (*p* = 0.24) [111].

## 5. Limitations

We acknowledge several limitations in this study. First, since some of the 100 mothers in this study were not part of the JA Kids study, information from the third trimester and delivery questionnaires were not complete. This resulted in missing information for some of the variables reported here. Additionally, maternal nutritional intake was not assessed, thus nutritional exposures to the metals examined here, which have previously been shown to be associated with blood concentrations of various metals [45,47,49,71] could not be investigated or adjusted for. Finally, there is the possibility of residual confounding by other factors not completely controlled for by the variables included in the analyses.

## 6. Conclusions

This study is the first to report levels of lead, mercury, arsenic, cadmium, manganese, and aluminum in cord blood of Jamaican newborns that could serve as a reference for the Jamaican population. In addition, the association of these metals in cord blood with certain birth outcomes was investigated. Our results indicate that cord blood lead concentrations in Jamaican newborns are significantly associated with their head circumference, after controlling for maternal education level and SES (through ownership of a family car). Our results also replicate previously reported significant associations between cord blood lead concentrations and birth head circumference in other populations. However, no such associations for concentrations of any other of the five trace elements/metals with birth outcomes were found. In the process of identifying factors associated with cord blood metal concentrations, we found significantly higher geometric mean cord blood aluminum concentrations in newborns whose mothers had a lower level of formal education as well as those who were born to families with lower SES. Therefore, improving SES and educating mothers regarding sources of exposure to aluminum could potentially reduce levels of this toxin in Jamaican newborns.
